# Sensitivity to airborne sounds in ice-dependent bearded seals

**DOI:** 10.1007/s00359-025-01782-1

**Published:** 2025-12-05

**Authors:** Noah Packard, Jillian M. Sills, Ryan A. Jones, Seri T. Aldana, Colleen Reichmuth

**Affiliations:** 1 Department of Ocean Sciences, University of California Santa Cruz, Santa Cruz, CA USA; 2 Institute of Marine Sciences, Long Marine Laboratory, University of California Santa Cruz, Santa Cruz, CA USA; 3 Alaska SeaLife Center, AK Seward, USA

**Keywords:** Pinniped, Phocid, Hearing, Masking, Critical ratio, Threshold

## Abstract

As the Arctic warms and becomes more accessible to human activities and associated noise sources, it is important to understand the auditory capabilities of ice-associated marine mammals that rely on sound. In this study, the in-air hearing of one adult bearded seal (*Erignathus barbatus*) was measured in ambient outdoor conditions using psychophysical methods. 50% detection thresholds were measured for 10 frequencies (0.04–51.2 kHz) that extend across the subject’s hearing range. For low to mid frequencies (0.04–12.8 kHz), thresholds were constrained, or masked, by ambient noise. Thresholds obtained at higher frequencies (25.6–51.2 kHz) were sufficiently elevated above background noise to provide absolute measures of hearing sensitivity. These measurements reveal that the high-frequency roll-off for bearded seals is in alignment with available auditory data for related species despite more than 11 million years of evolutionary isolation. Further, the data collected at low and mid frequencies enable an unconventional estimation of critical ratios, which can be applied in masking models. Collectively, these findings for bearded seals listening for airborne sounds highlight auditory similarities within the northern clade of phocid Carnivores and improve predictions of potential noise effects for seals in changing Arctic soundscapes.

**Supplementary Information:**

The online version contains supplementary material available at 10.1007/s00359-025-01782-1.

## Introduction

Bearded seals (*Erignathus barbatus*, Erxleben 1777) represent the earliest diverging lineage of the Phocinae (or ‘northern’) subfamily of seals, separated from the other nine species by 11 to 17 million years (Árnason et al. 2006; Higdon et al. 2007; Fulton and Strobeck 2010; Park et al. 2024). They have a broad pan-Arctic distribution that is restricted to high latitudes and are strongly associated with the seasonal movements of drifting, dense pack ice (Burns 1981; Kovacs 2016; Gryba et al. 2021). Notably, bearded seals are among the most pelagic of the true (phocid) seals. They spend nearly all of their time (> 90%) in frigid, shallow waters (< 100 m) and only rarely haul out onto sea ice to rest and nurse their dependent pups (Cameron et al. 2010). As benthic foragers for primarily sessile, invertebrate prey, bearded seals possess highly developed facial vibrissae that provide exceptional tactile sensitivity in the dark, turbid waters beneath the sea ice (Marshall et al. 2006; Marshall 2016).

Bearded seals can be distinguished from related species by several ancestral features (see Reichmuth and Klein 2025). Their skulls are particularly stout and robust relative to their large body size (> 250 kg), an adaptation thought to support their suction-feeding strategy (Marshall et al. 2008; Kienle and Berta 2016; Kienle et al. 2018). Additionally, bearded seals possess a relatively large external auditory meatus that opens in air and closes by muscular control during submergence. While a dynamic ear opening is characteristic of the Phocinae subfamily, the bearded seal’s auditory meatus is the largest of this group – exceptionally so when compared to species within the Monachinae (or ‘southern’) subfamily of seals, where the external meatus has been evolutionarily reduced to a closed canal (Ruscher et al. 2021). The implications of this distinct auditory morphology on the perception of airborne sounds by bearded seals are unknown.

Male bearded seals produce elaborate underwater vocal displays during the breeding season. These songs have unusual qualities relative to those of other aquatic-breeding seals, and certainly, their sound production can be considered remarkable among other Carnivores. Bearded seal vocalizations, most often classified as trills, moans, and sweeps, include a variety of high-amplitude, frequency-modulated calls that range from < 0.1 to > 10 kHz and can be detected above background noise tens of kilometers away (Cleator et al. 1989; Van Parijs et al. 2001; MacIntyre et al. 2013). Their long-duration songs are often associated with stereotyped dive displays and may play a role in male advertisement or resource acquisition during the breeding season (Van Parijs et al. 2001). Less is known about the in-air vocal behavior of bearded seals. As in related species, acoustic signaling may play a role in mother–pup interactions above the water’s surface (Insley et al. 2003; Charrier and Casey 2022; Linossier et al. 2025), but such airborne calls have not been described.

Similar to other northern seals, bearded seals rely on sound reception both under water and in air. Behavioral audiograms describing auditory sensitivity as a function of frequency are available for six of the ten Phocinae species in at least one testing medium (see Southall et al. 2019). In water, hearing curves for harbor (*Phoca vitulina*), ringed (*Pusa hispida*), and spotted seals (*Phoca largha*) indicate that these species have sensitive underwater auditory capabilities, with a range of best hearing from approximately 0.3–60 kHz and peak sensitivity near 50 dB re 1 µPa (Kastelein et al. 2009; Reichmuth et al. 2013; Sills et al. 2014, 2015). Available data for these seals indicate similar sensitivity to airborne sounds across species, with a range of best hearing from 0.5−14 kHz and peak sensitivity near − 10 dB re 20 µPa (Reichmuth et al. 2013; Sills et al. 2014, 2015). Despite significant evolutionary, morphological, and ecological differences, bearded seals possess underwater hearing sensitivity similar to that of related species in both quiet and noisy conditions (Sills et al. 2020). However, the ability of bearded seals to perceive airborne sounds has never been investigated.

Here, we characterize the species-typical in-air hearing capabilities of bearded seals. Using behavioral methods, we evaluate the sensitivity of one individual listening for airborne sounds across the range of hearing in an outdoor testing environment. Data for this individual are validated as representative for the species using measures of underwater auditory sensitivity available for several individuals and comparative data for other seal species. Absolute sensitivity measurements are reported for higher frequencies and masked thresholds for lower frequencies. In the latter case, corresponding signal-to-noise ratios allow for estimation of key masking parameters necessary for predicting noise effects with standard models. These sensitivity and masking data for the phylogenetically isolated bearded seal enable comparisons with other northern seals and inform a broad understanding of hearing among phocid Carnivores.

## Materials and methods

### Subject

**Fig. 1:**
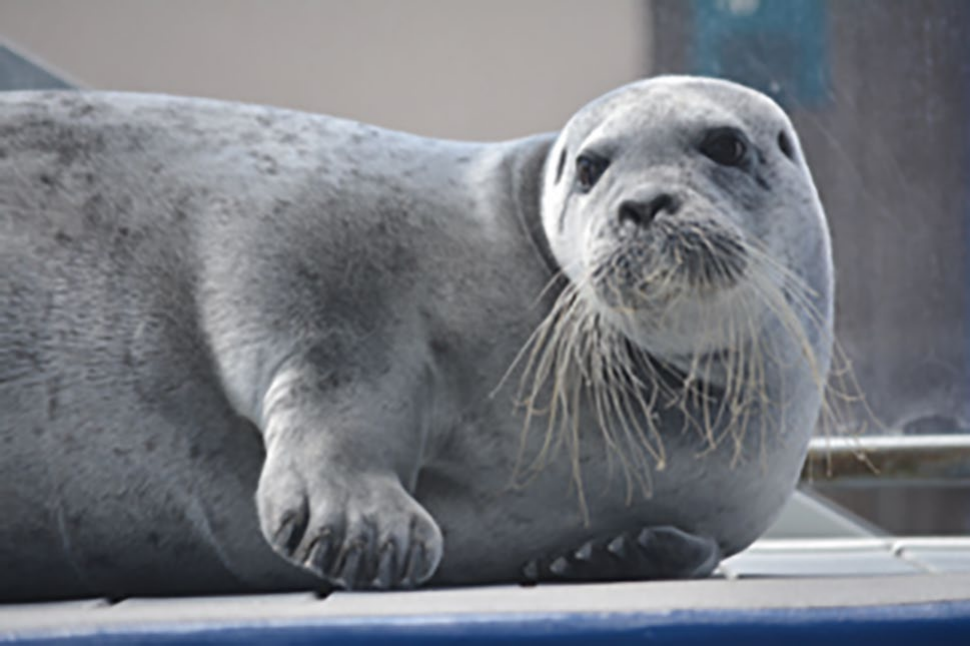
The bearded seal subject evaluated in the present study, demonstrating the distinct facial vibrissae, square-shaped flippers, and large auditory openings characteristic of the species. Photo: C. Reichmuth, NMFS 23554

Audiometric testing was conducted with an adult male bearded seal identified as *Noatak* (NOA0010270). This seal (Fig. 1) was 8–9 years old, had an intermeatal distance of approximately 18 cm, and weighed 202 to 244 kg throughout the testing period. The subject was in apparently good health, with no known otological problems or exposures to ototoxic medications.

*Noatak* was trained using operant conditioning and positive reinforcement for voluntary participation in husbandry and research procedures. He had previously participated in several psychoacoustic studies, including measurements of underwater hearing sensitivity and masking (Sills et al. 2020, 2025). The seal's diet was established to maintain optimal health and was not constrained for research purposes. He participated in 1 to 2 audiometric sessions per day, up to 5 days per week, and received about 15% of his daily diet for these sessions.

Authorization for this study was granted under US National Marine Fisheries Service marine mammal research permit 23554. Research protocols were reviewed and approved by the Institutional Animal Care and Use Committee at the University of California Santa Cruz, with expressed support from the Ice Seal Committee, a tribally authorized Alaska Native co-management organization.

### Environment and apparatus

Testing occurred outdoors at Long Marine Laboratory at the University of California Santa Cruz between November 2023 and July 2024. The test enclosure was a semi-enclosed 4 × 3 m triangular holding pen adjacent to the seal’s primary housing pool. The walls directly to the side and rear of the pen were synthetic high-density polyethylene (HDPE). The decking was composite material and the top of the pen was open to the outside environment but covered by shade cloth. During testing, the doors directly behind and to the left of the subject remained open to the adjacent pool.

The testing apparatus was a 0.3 × 0.4 m rectangular block of composite material placed in an open doorway at the front of the pen. A polyvinyl chloride (PVC) chin station was secured in the center of the block and a response target was mounted 0.1 m to the subject’s left when on station. While resting comfortably with his head on the chin station, the subject’s ears were 0.2 m above the deck. The speakers used to produce auditory stimuli were positioned in front of and on axis with the center of the chin station at distances of 0.8–1.1 m, depending on frequency.

Ambient noise measurements in the outdoor testing environment were taken daily in the position of the animal’s head under test-ready conditions. 1 min, unweighted noise samples were measured in 1/3-octave bands with a calibrated, self-powered 2250 sound level meter (sampling rate 48 kHz; Brüel & Kjær A/S, Nærum, Denmark) with a free-field 1/2-in. type 4966 microphone. Percentile statistics of these 1/3-octave band measurements were converted to units of power spectral density (PSD, dB re (20 µPa)^2^/Hz). High-frequency (25.6–51.2 kHz) ambient noise measurements were obtained twice during testing at each frequency using a MK301 microphone capsule (0.005–100 kHz; Microtech Gefell GmbH, Gefell, Germany) with a C617 body (Josephson Engineering, Santa Cruz, CA) and BPS-1 power supply (Stewart Electronics, Rancho Cordova, CA) linked to a battery-powered Fostex FR-2 Field Memory Recorder (Fostex Company, Tokyo, Japan).

### Stimulus generation and calibration

Test frequencies were 0.04, 0.05, 0.075, 0.4, 1.6, 3.2, 12.8, 25.6, 36.2, and 51.2 kHz. These frequencies were selected based on audiometric data collected from other phocid subjects (e.g., Sills et al. 2014). Audiometric signals were 0.5 s frequency-modulated linear upsweeps with 10% bandwidth and 25 ms linear ramps. They were generated (500 kHz update rate) from a laptop personal computer (PC) in LabVIEW (NI, Austin, TX) using Hearing Test Program (HTP) software (Finneran 2003). For frequencies below 25.6 kHz, signals passed through a NI USB-6251 data acquisition board and a two-channel Mix 2:1 passive mixer (Radial Engineering, Vancouver, Canada) before being projected through a KH80 DSP-powered studio monitor with an internal amplifier (0.057–21 kHz; Neumann, Berlin, Germany). For frequencies 25.6 kHz and above, signals were passed from the HTP software through the same data acquisition board, a 0.1–250 kHz bandpass active filter module (Krohn-Hite, Brockton, MA), the passive mixer, and a PA5 digital attenuator (Tucker-Davis Technologies, Alachua, FL, USA) before being projected through a Vifa ultrasonic dynamic speaker with a portable ultrasonic power amplifier (Avisoft Bioacoustics, Glienicke/Nordbahn, Germany).

The sound field of the test environment was spatially mapped prior to the start of data collection. Signals were received across 14 positions within a 4 cm x 4 cm x 4 cm area surrounding and including the position of each ear during testing. Variability in received sound pressure level (SPL) across all positions was required to be +/- 3 dB. Audiometric signals were also calibrated daily in the position of the ear that received the higher level during mapping. Signals for frequencies below 25.6 kHz were received by the Brüel & Kjær 2250 sound level meter. For frequencies 25.6 kHz and above, signals were received by the MK301 microphone capsule with Josephson C617 body and BPS-1 power supply. Received signals were passed through the same data acquisition board and measured in HTP. The received stimuli were inspected as waveforms and spectrograms to confirm signal integrity and expected narrow-band spectrum at the target frequency. The system was validated regularly with a RION NC-73 sound level calibrator.

### Audiometry

The auditory task was a go/no-go signal detection paradigm (Stebbins 1970) consisting of both signal-present and signal-absent trials (see Online Resource 1). At the start of each session, the seal was prompted by an assistant to enter the testing environment and rest his head on the chin station. During testing, the assistant remained to the side of the subject behind a plexiglass wall and was unaware of individual trial conditions. Correct detections on signal-present trials (touching the response target with his nose) or correct rejections on signal-absent trials (remaining on the station for the full 4 s trial duration) were marked with a conditioned acoustic reinforcer (buzzer or verbal bridge) followed by a primary reinforcer (fish) delivered near the response target. Missed detections on signal-present trials and false detections on signal-absent trials were not reinforced. Instead, the subject was recalled by name and re-prompted to the chin station before proceeding to the next trial. Sessions were controlled remotely from a sound-isolated room, where the operator monitored the subject in real-time using a mounted video camera and provided feedback to the assistant through headphones.

Using an adaptive staircase method, signal frequency remained constant within a session while signal amplitude was adjusted based on subject performance (Stebbins 1970; Cornsweet 1962). Following the first, easily detectable signal-present trial, amplitude was decreased by 2–4 dB after each correct detection until the subject missed. Amplitude was then increased by 4 dB after each miss and decreased by 2 dB after each correct detection until at least 3 descending misses (hit-to-miss transitions) were obtained. Trial order was pseudorandomly established according to a set ratio, with signals presented on 55–60% of trials during an approximately 40-trial session. False alarm rates were determined for each session as the proportion of false detections to signal-absent trials.

### Threshold determination

Frequencies were tested to completion across successive days with frequencies evaluated in a shuffled order. Hearing threshold at each frequency was calculated as the average of 15 stable hit-to-miss transitions over 2–3 sessions with a standard deviation of < 3 dB and a pooled false alarm rate > 0 and < 0.3. Threshold-to-noise offsets were calculated as the difference between measured hearing threshold and ambient noise spectral density level at each frequency.

To evaluate a possible gain in audibility with increased signal duration at low frequencies, hearing at 0.05 kHz was remeasured at the end of the experiment. The equipment chain and session parameters remained the same, while signal duration was increased from 0.5 s to 1 s.

### Consideration of noise influence on threshold measurements

To determine whether environmental noise constrained hearing at any test frequencies, measured thresholds were assessed in relation to the ambient noise of the testing environment. The masking parameter most commonly applied to evaluate the effects of noise on signal detectability is the critical ratio. This frequency-specific signal-to-noise ratio is the amount by which a signal must exceed temporally overlapping noise of similar spectral content in order to be detected (Fletcher 1940). The bearded seal’s actual or extrapolated critical ratio values (Jones 2024; Sills et al. 2025) were added to ambient noise spectral density levels to estimate the theoretical lowest thresholds measurable in the outdoor testing environment. Thresholds that fell above these theoretical lowest values were considered to reflect absolute hearing sensitivity, while those that fell near or below these theoretical lowest thresholds were likely constrained by background noise. As ambient noise measurements were limited by the electrical noise floor at frequencies above 20 kHz, minimum estimated threshold-to-noise offsets were instead used to conservatively determine whether thresholds were masked in the outdoor testing environment.

## Results

**Fig. 2:**
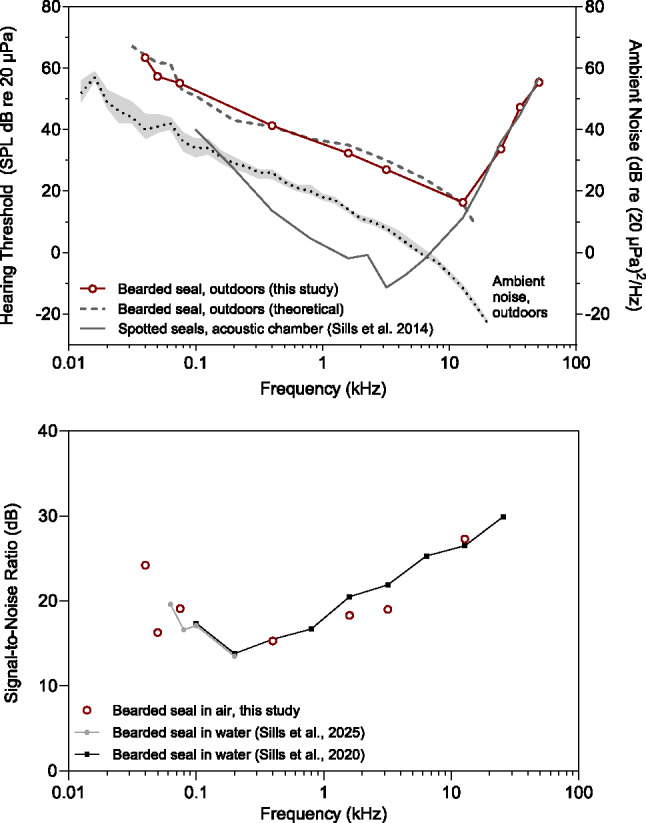
In-air hearing data for bearded seal *Noatak*. The upper panel plots hearing thresholds (open circles) at 10 frequencies tested in outdoor ambient conditions using psychophysical methods. The median noise levels in this testing environment are plotted corresponding to the right-hand y-axis. The 50th percentile levels are shown as a dotted line bounded (shaded region) by the 10th (above) and 90th (below) percentile statistics of the noise distribution. Corresponding theoretical lowest threshold values for this individual in this environment are plotted as a dashed line based on measured noise and critical ratio data (see text for details). For comparison, previously published in-air hearing data for a representative northern seal species (spotted seal, *Phoca largha*) are provided as a solid line (Sills et al. 2014; *n* = 2). The lower panel depicts signal-to-noise ratios (open circles) associated with the bearded seal's measured hearing thresholds at the seven frequencies determined to be constrained (masked) by background noise in this testing configuration (see Table 1). These signal-to-noise ratios can be considered estimates of critical ratio values. Available critical ratio data obtained previously for the same individual in water are also shown

Ambient noise in the outdoor testing enclosure was highest at low frequencies and declined with increasing frequency (Table 1; Fig. 2). Median noise levels ranged from 52 dB re (20 µPa)^2^/Hz at 0.013 kHz to −23 dB re (20 µPa)^2^/Hz at 20 kHz. At frequencies from 20 to 60 kHz, ambient noise values were limited by the measurement system; however, noise spectral density levels were below 0 dB re (20 µPa)^2^/Hz. Thus, 0 dB re (20 µPa)^2^/Hz was conservatively considered the ambient noise level for test frequencies of 25.6, 36.2, and 51.2 kHz.

**Table 1:** In-air hearing thresholds obtained for one bearded seal using psychophysical methods

Frequency kHz	Threshold dB re 20 µPa	Standard deviation	False alarm rate	Ambient noise dB re (20 µPa)^2^/Hz	Theoretical lowest threshold dB re 20 µPa	Threshold-to-noise offset dB	Published critical ratio* dB	Masked or unmasked
0.04	64	1.3	0.12	40	64	24	-	Masked
0.05	57	2.1	0.09	41	62	16	-	Masked
0.075	55	1.4	0.13	36	54	19	-	Masked
0.4	41	2.3	0.15	26	41	15	15	Masked
1.6	32	1.5	0.11	14	35	18	21	Masked
3.2	27	1.3	0.17	8	30	19	22	Masked
12.8	16	1.6	0.29	−11	16	27	27	Masked
25.6	34	1.6	0.13	< 0	-	> 34	30	Unmasked
36.2	47	2.0	0.06	< 0	-	> 47	-	Unmasked
51.2	55	1.7	0.11	< 0	-	> 55	-	Unmasked

In-air hearing thresholds for the bearded seal (*Noatak*), with corresponding standard deviations, false alarm rates, ambient noise levels in the testing environment, theoretical lowest thresholds, threshold-to-noise offsets, previously published critical ratios, and indications of whether each threshold was likely masked or unmasked. Detection thresholds at each frequency are the average of 15 hit-to-miss transitions. False alarm rates are the proportion of responses on signal-absent trials (> 45 trials per frequency). Ambient noise is provided in terms of spectral density levels calculated from the median of measured 50th percentile 1/3-octave band levels across all testing days. Theoretical lowest thresholds for 0.04, 0.05, and 0.075 kHz are based on extrapolated critical ratio data (Sills et al. 2025)

*Additional critical ratio data for this bearded seal are available and are reported in Fig. 2 (Sills et al. 2020, 2025)

Hearing thresholds for the narrowband airborne stimuli were collected at ten frequencies from 0.04 to 51.2 kHz (Table 1; Fig. 2). The lowest measured threshold was 16 dB re 20 µPa at 12.8 kHz. The highest measured threshold was 64 dB re 20 µPa at 0.04 kHz. Mean false alarm rate throughout testing was 0.14 (range 0.06–0.29). Threshold-to-noise offsets, measured as the difference between the seal’s hearing threshold and ambient noise, were 16 to 27 dB and greatest at high frequencies.

Comparisons of threshold-to-noise offsets and actual or extrapolated critical ratios (Table 1; Sills et al. 2020; Sills et al. 2025) confirmed that thresholds at 25.6 kHz and above were sufficiently elevated above background noise to provide absolute measures of hearing sensitivity. Thus, the high-frequency roll-off was accurately captured for this individual. Following the inflection point at 12.8 kHz, sensitivity decreased by ~ 20 dB per octave to 51.2 kHz, the highest frequency tested. Extrapolation above this frequency indicated that the high-frequency hearing limit – defined by Heffner and Heffner (2008) as the highest frequency audible at 60 dB re 20 µPa – was 52.7 kHz. The high-frequency roll-off of this bearded seal was comparable to available data for other northern seals tested in much quieter conditions (Reichmuth et al. 2013; Sills et al. 2014, 2015). Relative to terrestrial Carnivores, this bearded seal’shigh-frequency hearing sensitivity is most similar to that of dogs and ferrets, with a high-frequency hearing limit somewhat lower than cats and the least weasel (Heffner 1983; Heffner and Heffner 1985a, b; Kelly et al. 1986).

Between 0.04 and 12.8 kHz, ambient thresholds were well predicted by theoretical lowest thresholds in this testing environment (within 2 dB, on average), indicating that measured thresholds were constrained by ambient noise (Table 1; Fig. 2). Therefore, reported values provide only an upper limit on bearded seal absolute hearing thresholds at these frequencies. On the low-frequency roll-off (< 100 Hz), masked threshold values less than 65 dB suggest hearing sensitivity is comparable to or better than that of terrestrial Carnivores. (Heffner 1983; Heffner and Heffner 1985a, b; Kelly et al. 1986).

While thresholds at 12.8 kHz and below do not reveal absolute sensitivity, these data do provide useful information about hearing in noise. The threshold-to-noise offsets at these frequencies can be considered estimates of critical ratios, as they indicate the amount (in dB) by which the signal must exceed surrounding background noise to be detected. These measured signal-to-noise ratios are plotted in Fig. 2 alongside published critical ratio data for the same bearded seal. Using this unconventional method, critical ratios can be estimated at lower frequencies than have been tested previously for the species. Predicted low-frequency critical ratios are 24 dB at 0.04 kHz, 16 dB at 0.05 kHz, and 19 dB at 0.075 kHz. At the higher test frequencies for which critical ratios were already available (0.4, 1.6, 3.2, and 12.8 kHz; Sills et al. 2020), the in-air signal-to-noise ratios from this study fell within 3 dB of those critical ratios obtained in water.

Repeated testing at 0.05 kHz with signal duration of 1 s revealed a difference in measured threshold of 1 dB (57 dB at 0.5 s and 56 dB at 1 s), confirming no gain in audibility with increased duration beyond 0.5 s. These results are consistent with some previous measures of low-frequency temporal processing in seals (Reichmuth et al. 2012; Sills et al. 2025) and confirm that 0.5 s is an appropriate signal duration for testing hearing at the low frequencies evaluated in this experiment.

## Discussion

### Sensitivity to airborne sounds

Hearing thresholds measured for the bearded seal in this study provide the first measures of sensitivity to airborne sounds in this species. While characterization of absolute sensitivity at lower frequencies was limited by background noise in the outdoor testing environment, bearded seals can detect sounds at least as low as 0.04 kHz with a threshold  ≤ 63 dB re 20 µPa. Conservatively, the bearded seal’s hearing range extends from < 0.05 kHz to 53 kHz. In order to confirm absolute hearing sensitivity at and below 12.8 kHz, testing would need to be completed in a quieter, acoustically controlled environment, such as the acoustic chamber used in prior studies of in-air hearing in related species (see Sills et al. 2014). Nonetheless, at and above 25.6 kHz, reported absolute thresholds confirm the high-frequency hearing roll-off is consistent between bearded seals and other northern phocids. Despite differences in evolutionary history, behavior, and anatomy that isolate bearded seals from their closest relatives—particularly the stout skull, large meatal openings, and greater interaural distance—auditory data confirm that bearded seals share sensitive high-frequency hearing capabilities with related species.

### Masking by broadband noise

While measured thresholds at 0.04 to 12.8 kHz were constrained by ambient noise, the associated signal-to-noise ratios in this environment provided an opportunity to predict critical ratios. Critical ratios are typically measured as the difference (in dB) between the sound pressure level of a tonal signal at threshold and the power spectral density level of controlled, spectrally flat masking noise centered on the tone frequency (Fletcher 1940). While not true critical ratios, the signal-to-noise ratios collected here in relatively continuous broadband background noise do similarly indicate the amount by which a signal must exceed the noise backgroundto be detected. When compared to available critical ratios for this bearded seal, the signal-to-noise ratios from this study fell within 3 dB of published values. This finding confirms this method provides a reasonable estimation for critical ratios when measurements in more controlled conditions are unavailable or impossible. This masking methodology has been validated by similar studies with California sea lions and walruses (Jones et al. 2023).

In this case, using the signal-to-noise ratio at masked threshold to predict critical ratio allows for this key masking parameter to be estimated at lower frequencies than have been measured previously for any seal species (Erbe et al. 2016; Branstetter and Sills 2022; Sills et al. 2025). Critical ratios estimated for the bearded seal at 0.04, 0.05, and 0.075 kHz in this study are generally consistent with linear extrapolation from available data for the same species in water at 0.063 to 0.2 kHz (Sills et al. 2020, 2025). The lower-than-expected signal-to-noise ratio at 0.05 kHz could not be attributed to deviation in subject performance, as we observed similar thresholds and variance in repeated testing with 0.5 and 1.0 s signals, or to fluctuations in background noise, as there was no particular variability near this frequency in the repeated noise floor measurements.

Overall, the estimated low-frequency critical ratios from this study demonstrate that these masking values increase with decreasing frequency below about 0.2 kHz, consistent with available data for seals (Branstetter and Sills 2022; Sills et al. 2025). As critical ratios are a cochlear phenomenon and independent of medium (see Reichmuth 2012), our findings inform the application of masking models for seals listening for both airborne and underwater sounds in the presence of natural and human-generated noise.

### Species-typical hearing abilities

While the measurements reported here are for a single individual, these data can be considered representative for the species. This individual previously participated in auditory testing in water, and his hearing sensitivity aligned almost perfectly with that of two other bearded seals trained for the same cooperative sensory assessments (Sills et al. 2020, 2025). Furthermore, the absolute hearing thresholds determined for this subject in air at relatively high frequencies are the same as those reported for other Phocinae species, including harbor, spotted, and ringed seals (Reichmuth et al. 2013; Sills et al. 2014, 2015). Thus, the findings are both plausible and parsimonious, and unlikely to be biased by subject effects. From an applied perspective, the bearded seal’s absolute (unmasked) thresholds are aligned with the Phocid Carnivores in Air (PCA) hearing group proposed in recent marine mammal noise exposure criteria (National Marine Fisheries Service 2024), indicating the PCA weighing function is likely appropriate for predicting noise effects for this species.

As the evolutionary outgroup of the Phocinae subfamily of phocid Carnivores, consistency in amphibious hearing capabilities between the bearded seal and related species lends further support to the conclusion that northern seals comprise a unified functional hearing group, which may be conservative for the broader true seal lineage based on available data (Ruscher et al. 2025).

## Supplementary Information

Below is the link to the electronic supplementary material.

Supplementary Material 1.

## Data Availability

All data supporting the findings of this study are available within the paper and its Supplementary Information.
